# The role of m6A methylation in therapy resistance in cancer

**DOI:** 10.1186/s12943-023-01782-2

**Published:** 2023-06-01

**Authors:** Hengzhao Zhuang, Bo Yu, Dan Tao, Xiaoyan Xu, Yijun Xu, Jian Wang, Yang Jiao, Lili Wang

**Affiliations:** 1 Department of Radiation Oncology, The First Affiliated Hospital of Soochow University, Suzhou, 21500 China; 2 Department of Radiotherapy, The Affiliated Jiangyin People’s Hospital of Nantong University, Jiangyin, 214400 China; 3 Department of Radiation Oncology, Dushu Lake Hospital Affiliated to Soochow University, Suzhou, 21500 China; 4 School of Radiation Medicine and Protection, Medical College of Soochow University, Suzhou, 215000 China

**Keywords:** m6A methylation, Cancer, Chemoresistance, Radiotherapy resistance

## Abstract

Cancer therapy resistance is the main cause of cancer treatment failure. The mechanism of therapy resistance is a hot topic in epigenetics. As one of the most common RNA modifications, N6-methyladenosine (m6A) is involved in various processes of RNA metabolism, such as stability, splicing, transcription, translation, and degradation. A large number of studies have shown that m6A RNA methylation regulates the proliferation and invasion of cancer cells, but the role of m6A in cancer therapy resistance is unclear. In this review, we summarized the research progress related to the role of m6A in regulating therapy resistance in cancers.

## Introduction

Cancer is a major public health problem in the world. According to the data from the National Cancer Center (China) published in March 2022, there were approximately 4 million new cancer cases and 2.4 million cancer-related deaths in China in 2016 [1]. In the United States, approximately 0.59 million people died of cancer in 2019, accounting for 21% of all deaths. Cancer has become the second leading cause of death in the United States after heart disease [2]. In addition to traditional treatment methods including surgery, radiotherapy and chemotherapy, the main emerging treatment methods for cancer include immunotherapy, targeted therapy, and gene therapy. Currently, comprehensive and individualized therapy is regarded as the most common and promising method for cancer treatment [3, 4].

**Fig. 1:**
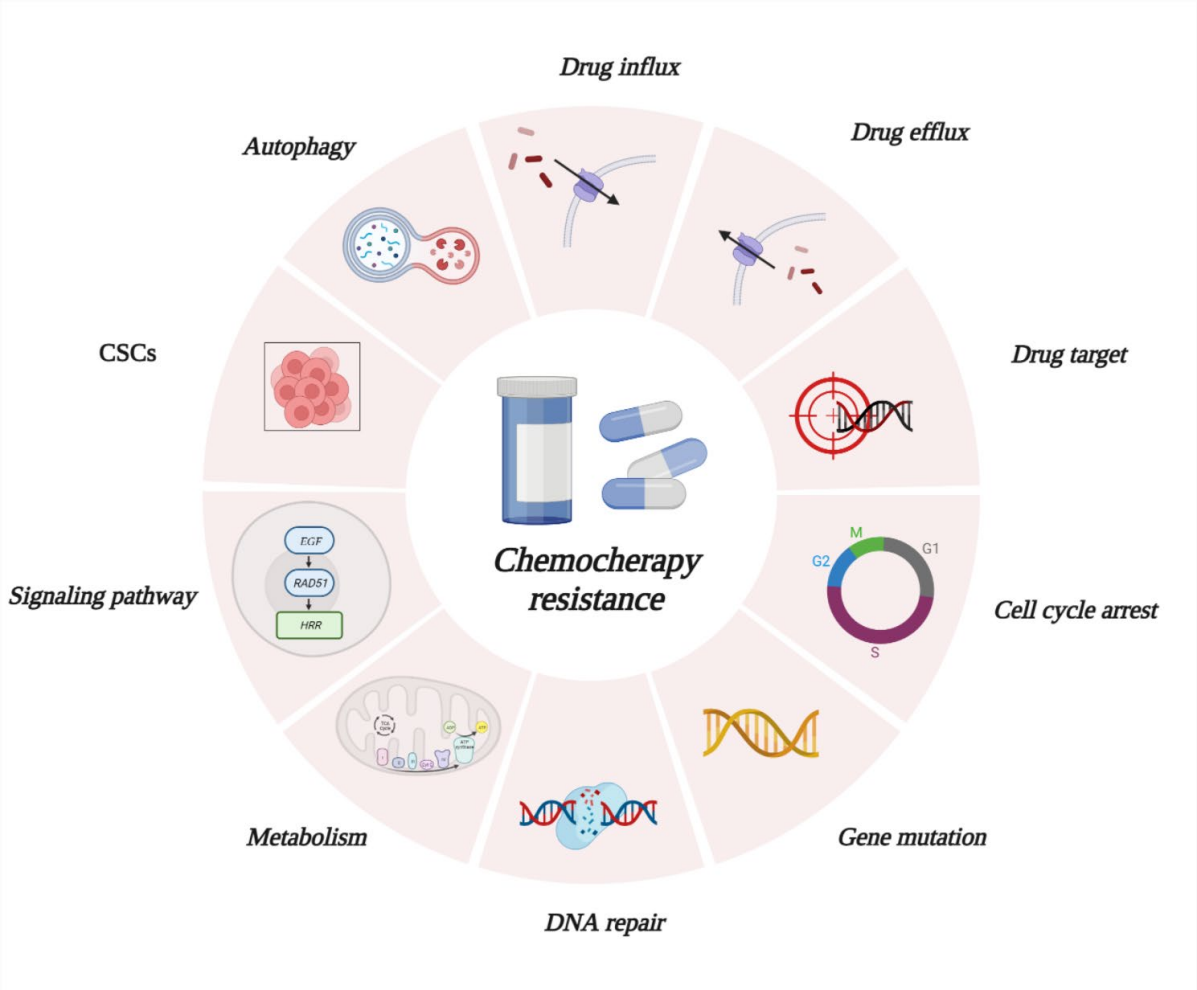
The mechanisms of chemical drugs resistance Increased drug efflux and decreased drug influx; Secondary mutation in drug target; Cell cycle arrest; Gene mutation and DNA damage repair; Changes in metabolism and signaling pathway; Generation of cancer steam cells; Inducing autophagy

The main reason for cancer treatment failure is that patients are resistant to treatments such as chemotherapy, which leads to tumor recurrence, progression, or distant metastasis. Chemotherapy drugs can effectively kill fast-growing tumor cells, which are widely used in the treatment of various tumors. However, there are still patients with a poor prognosis, and approximately 90% of cases of treatment failure are related to drug resistance [5]. Patients can present resistance to a specific drug or to multiple drugs with different structures and mechanisms, which is defined as multidrug resistance (MDR). Chemotherapy drug resistance is divided into intrinsic resistance and acquired resistance [6]. Intrinsic resistance refers to innate resistance that exists before patients are exposed to drugs. It is related to inherent genetic mutations in tumors. For example, patients suffering from gastric cancer with HER2 (human epidermal growth factor receptor 2) upregulation have an inferior response to cisplatin [7]. Acquired resistance refers to diminishing response to drugs after treatment, and secondary mutations in drug targets is one of the explanations. For instance, BCR-ABL fusion gene is a target for imatinib, a tyrosine kinase inhibitor, which is widely used in the treatment of chronic myeloid leukemia. If threonine 315 in its kinase domain is mutated, the binding ability of imatinib to BCR-ABL will decrease, thus significantly reducing drug efficacy. Approximately 20–30% of patients do not have a complete cytogenetic response following completion of imatinib [8]. In addition, cancer cell resistance to chemotherapy occurs at other levels, including increased drug efflux; decreased drug influx; cancer steam cells (CSCs); autophagy and so on(Fig. 1).

**Fig. 2:**
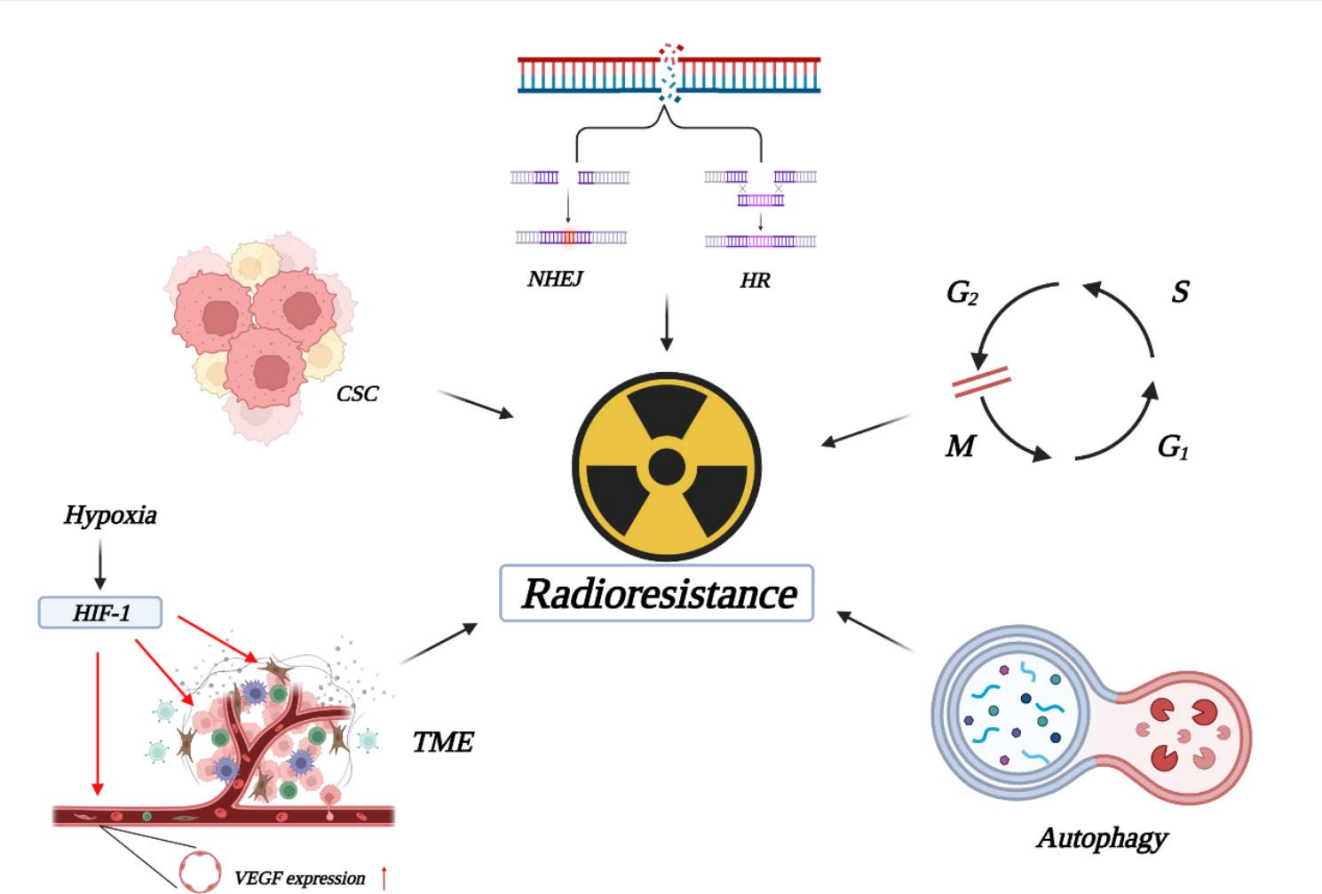
The mechanisms of radiotherapy resistance Nonhomologous end joining (NHEJ) and homologous recombination (HR); G2/M arrest and the accumulation of a large number of cells in S phase; Autophagy inhibits the production of reactive oxygen species; Changes in the tumor microenvironment and hypoxia; Generation of cancer stem cells

Apart from drug resistance, radiation resistance is another cause of treatment failure. Radiation resistance refers to the adaptation of tumor cells or tissues to radiotherapy. It is a complex process involving multiple genes and various mechanisms. The main reasons are as follows: ① DNA damage repair: DNA damage occurs after cells are irradiated, which is one of the reasons why radiation therapy is applied for tumor treatment. DNA damage repair is one of the causes of radiation resistance, which involves several pathways. For example, the PI3K (Phosphoinositide 3-kinase) signaling pathway is an important signaling pathway that facilitates DNA double-strand breaks (DSBs) repair by regulating nonhomologous end joining (NHEJ) and homologous recombination (HR) [9]. PI-103, the PI3K inhibitor, enhances radiation-induced cell death significantly [10]. ② Cell cycle arrest: After the occurrence of DNA damage, cell cycle arrest might occur, thus providing time for repair. Radiation induces G2/M phase arrest, accompanied by accumulation of a large number of cells in S phase, and the cells in S phase are resistant to radiation [11–14]. ③ Generation of CSCs: CSCs are undifferentiated cancer cells with high tumorigenic activity, self-renewal ability and multidirectional differentiation potential. It has been suggested that this mechanism may be related to the vascular wall. In brain tumors, endothelial cells can secrete certain factors that keep CSCs in a self-renewing and undifferentiated state and protect them from radiation damage, thus promoting tumor radiation resistance [15]. Compared with tumors in wild-type mice, tumors in mice with altered endothelial cells are resistant to apoptosis induction by radiation over 20 Gy [16]. ④ Changes in the tumor microenvironment (TME) and hypoxia. TME consists of a chemical microenvironment (characterized by pH, NO and metabolites, such as glucose) and a cellular microenvironment including tumor cells, stromal cells and extracellular matrix (ECM) [17]. Cancer-associated fibroblasts (CAFs) are dynamic components of the TME that affect the occurrence and development of tumors by producing ECM proteins, secreting growth factors and regulating the epithelial-mesenchymal transition [18]. Hypoxia promotes tumor progression by regulating CAFs function. Hypoxia can activate hypoxia-inducible factor-1 (HIF-1) and stimulate the expression of transforming growth factor-β (TGF-β), promoting fibroblast activation. Hypoxia also increases the expression of vascular endothelial growth factor in CAFs, which produces endothelial cells radioresistance and promotes the proliferation and regeneration of tumor vessels [19, 20]. ⑤ Autophagy: Autophagy, which occurs in almost all eukaryotic cells, is a highly evolutionarily conserved physiological process. By degrading and making use of long-lived proteins and cytoplasmic organelles, autophagy plays an important role in maintaining intracellular metabolic homeostasis [21]. Under stresses such as hypoxia, inadequate growth factors, radiation or chemical drugs, tumor cells can escape from death via autophagy-mediated inhibition of the apoptotic pathway, thereby inducing the development of drug resistance [22]. Moreover, autophagy inhibits the production of reactive oxygen species to enhance radiation resistance of cells [23](Fig. 2).

Cancer therapy resistance is a complex process, and understanding its molecular mechanisms can help us overcome it. Epigenetics plays an important role in cancer treatment resistance. Progress in research on DNA methylation, histone modification, chromatin remodeling and RNA modification has led to a better understanding of therapy resistance. For example, DNA demethylation in the promoter region of oncogenes increases their expression, leading to drug resistance. Thymosin β4 (Tβ4) is abnormally expressed after DNA demethylation and histone H3 modification in the promoter region. Overexpression of Tβ4 enables hepatoma carcinoma cells to acquire cancer stem cell-like abilities and causes resistance to sorafenib [24]. Although research on RNA modification began as early as the 1970s, it has been stalled by technical problems. In 2012, a novel approach combining RNA immunoprecipitation with next-generation sequencing was developed and allows further study of RNA modifications [25]. RNA methylation accounts for more than 60% of RNA modifications. The 5’ cap and 3’ poly-A modifications in eukaryotic cell mRNA play a key role in transcriptional regulation, while the internal modifications of mRNA usually maintain mRNA stability, such as N6-adenylate methylation and N1-adenylate methylation [26]. N6-methyladenosine (m6A) is one of the most common internal modifications in eukaryotic cells and has an important impact on mRNA splicing, transport, translation and other processes [27].

**Fig. 3:**
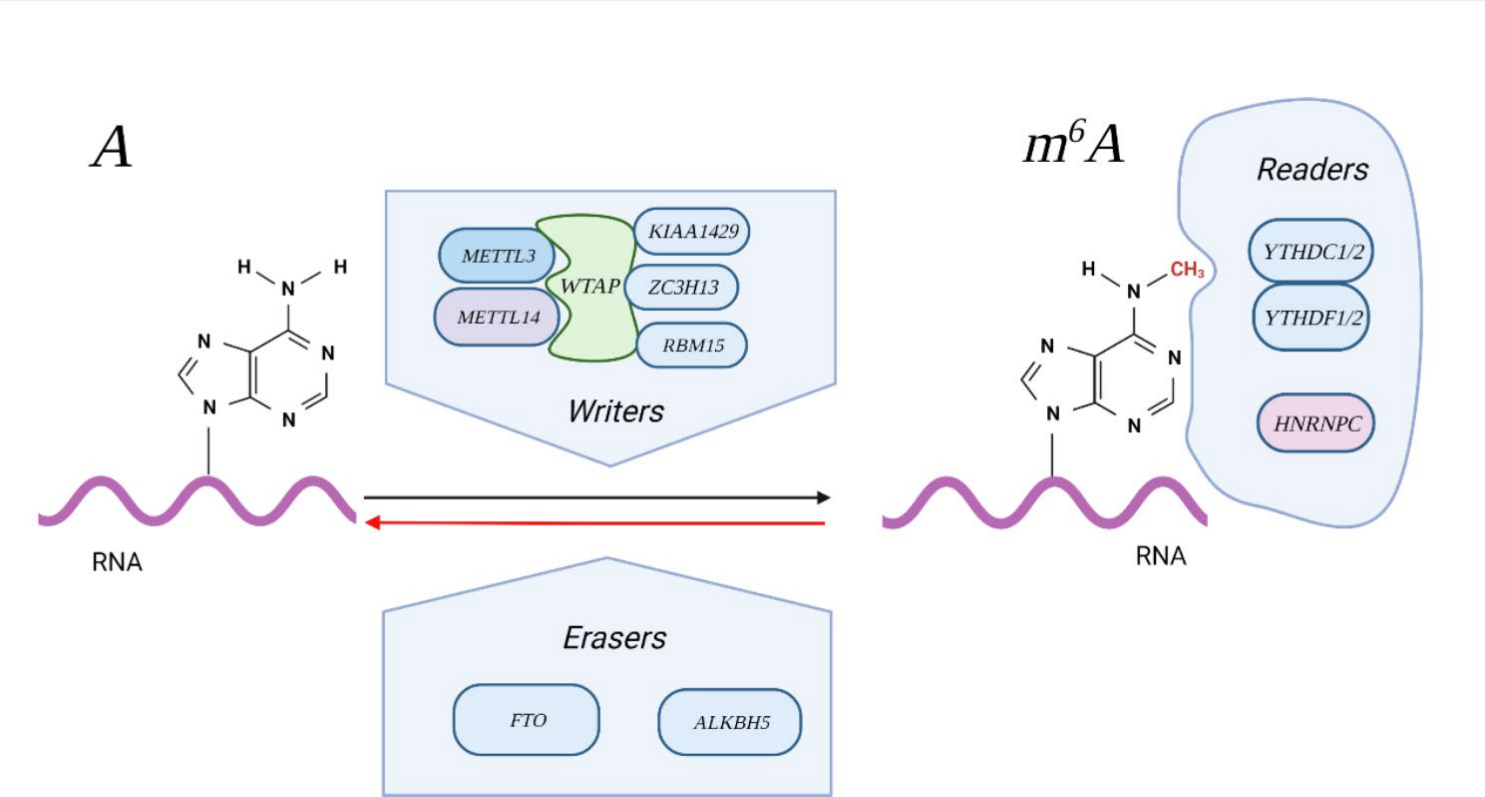
The process of m6A methylation The “writers” add methyl groups to RNA. METTL3 and METTL14 have a substrate recognition function. WTAP is responsible for recruiting METTL3, METTL14 and other components. The m6A modification is removed by “erasers” (FTO and ALKBH5). The “readers” recognize m6A and affect various functions of RNA, and they mainly include members of the YTH domain-containing family (YTHDC1/2 and YTHDF1/2) which can degrade mRNA.

The m6A methylation is a dynamic reversible process regulated by three factors. Methyltransferases, called “writers”, include METTL3, METTL14, WTAP, RBM15, ZC3H13, and KIAA1429 (VIRMA). METTL3 is a core subunit with catalytic activity and METTL14 has a substrate recognition function. WTAP is responsible for recruiting METTL3 and METTL14 and combining with other components to form hybrids. “Erasers”, including FTO and ALKBH5, can demethylate RNA. FTO has the similar structure with ALKBH5 in core domain and is closely related to obesity and cancer. Methylated RNA is recognized by different enzymes to perform specific biological functions. The proteins that recognize methylation sites are called “readers”, that include YTHDC1, YTHDC2, YTHDF1, YTHDF2, and HNRNPC. Proteins with the YTH domain bind to methylated mRNA specifically and regulate downstream translation and degradation(Fig. 3).

A growing number of studies suggest that m6A methylation is a broad regulatory mechanism of gene expression. However, the role of m6A in the mechanism of cancer treatment resistance is unclear. Herein, we focus on the mechanisms of therapeutic resistance associated with m6A RNA methylation and potential approaches to overcome it.

## Chemoresistance and m6A methylation

### Cisplatin

The antibacterial activity of cisplatin was first discovered in 1965. Subsequently, it was observed to have a powerful antitumor effect, cross-linking with DNA to disrupt its normal function. In 1978, cisplatin was approved as an antitumor drug for clinical use, and approval of carboplatin and oxaliplatin were followed. Although the current theme of cancer treatment is precision targeting and immunotherapy, platinum-based drugs are still one of the most widely used antitumor drugs because of its significant and broad spectrum of antitumor activity [28]. Many studies have indicated that m6A is associated with the development of cisplatin resistance in tumors. HOXA10 is a member of the HOX gene family. The “erasers” ALKBH5 and HOXA10 form a positive feedback loop, which eliminates the m6A modification of JAK2 mRNA and promotes cisplatin resistance in epithelial ovarian cancer by activating the JAK2/STAT3 signaling pathway. Cell sensitivity to cisplatin was rescued by ALKBH5 and HOXA10 knockdown or inhibition of the JAK2/STAT3 signaling pathway [29]. Similarly, ALKBH5 reduces the stability of casein kinase (CK) 2α mRNA via demethylation, resulting in a decrease in CK2α protein expression. Casein kinase 2 (CK2) is a serine/threonine kinase that affects glycolysis in cancer cells. Research has reported that ALKBH5 was downregulated in bladder cancer cells. Knockdown of ALKBH5 promotes the proliferation and migration of bladder cancer cells, which may be achieved by increasing glucose utilization, lactate production and intracellular ATP levels in cancer cells. Moreover, ALKBH5 overexpression can enhance the sensitivity of bladder cancer cells to cisplatin through the CK2α-mediated m6A-dependent glycolysis pathway [30, 31]. Cisplatin resistance caused by ALKBH5 is not only associated with metabolic pathways but is also with CSCs. It has been found that the RNA helicase DDX3 mediated cisplatin resistance in oral squamous cell carcinoma (OSCC) by regulating the expression of the CSCs transcription factors FOXM1 and NANOG through ALKBH5. Pharmacological (ketorolac salt) inhibition of DDX3 restored cisplatin-mediated cell death and reduced CSCs population. A combination regimen of ketorolac salt with cisplatin may rescue OSCC resistance to chemotherapy [32]. It suggested that the development of cisplatin resistance is caused by the eraser-mediated demethylation process that destabilizes the mRNA of certain key enzymes. In fact, “writers” as well contribute to this process. One study showed that circ0008399 binding to WTAP promoted TNFα-induced protein 3 (TNFAIP3) expression by increasing its mRNA stability in an m6A-dependent manner, reducing the chemosensitivity of bladder cancer cells to cisplatin. The targeted regulation of the circ0008399/WTAP/TNFAIP3 axis could enhance cisplatin efficacy [33]. In nasal-type natural killer/T-cell lymphoma (NKTCL), WTAP stabilizes DUSP6 mRNA and promotes DUSP6 protein expression, which results in cisplatin resistance [34]. DUSP6, a dual-specificity phosphatase, is a negative regulator of the RAS-ERK signaling pathway and plays a key role in tumor progression, invasion, metastasis and chemoresistance [35]. In addition to WTAP, METTL3 is related to cisplatin resistance. Chen et al. found that METTL3 induced autophagy by regulating ATG5 expression, which enhanced seminoma resistance to cisplatin. The use of autophagy inhibitor 3-methyladenine (3-MA) and knockdown of ATG5 could overcome the cisplatin resistance in seminoma [36]. Wei et al. found that METTL3 rendered seminoma insensitive to cisplatin by stabilizing TFAP2C mRNA and activating the DNA repair-gene BRCA1 [37]. KIAA1429 (VIRMA) regulated the response of seminoma to cisplatin by disturbing DNA damage, and tumor sensitivity to cisplatin was increased after KIAA1429 knockdown [38]. In pancreatic cancer, METTL3 modulates the MAPK cascade to increase the resistance of pancreatic cancer cells to chemotherapy and radiotherapy [39]. Moreover, “readers” have also contributed to the development of cisplatin resistance. It has been reported that YTHDF1 promoted the production of GLS1, a key enzyme in glutamine metabolism, and regulated the glutamine metabolic pathway in colon cancer cells to make them resistant to cisplatin [40]. In ovarian cancer, YTHDF1 enhances the resistance of ovarian cancer cells to cisplatin, which may result from the maintenance of ovarian cancer CSCs through interaction with TRIM29. Hao et al. found that knockdown of YTHDF1 significantly reduced TRIM29 expression and suppressed stem cell-like features of ovarian cancer cells [41]. The CDKN1B gene encodes p27 protein, a cyclin-dependent kinase inhibitor, which arrests cell cycle in the G1 phase. One study showed that p27 can enhance the DNA damage response [42]. The cytotoxic effects of cisplatin are also dependent on DNA damage. YTHDF2 promotes the progression of intrahepatic cholangiocarcinoma and decreases its sensitivity to cisplatin by decreasing CDKN1B mRNA expression [43]. Wu et al. found that YTHDF2 knockdown inhibited the epithelial-mesenchymal transition process of cervical cancer cells and that YTHDF2 and AXIN1 contributed to cisplatin resistance in cervical cancer cells [44].

### Anthracyclines

Since the 1970s, anthracyclines have been regarded as the most effective chemotherapy drugs for breast cancer and are widely used in neoadjuvant therapy or combination therapy [45]. However, drug resistance to anthracyclines frequently occurs, leading to the recurrence and metastasis of tumors. Research has shown that m6A methylation is associated with the development of chemoresistance in breast cancer. miR-221-3p is a miRNA involved in tumorigenesis, metastasis, and drug resistance. METTL3 methylates pri-miR-221-3p mRNA to increase miR-221-3p expression and negatively regulate HIPK2, a tumor suppressor that can be activated by doxorubicin. Therefore, METTL3 may make breast cancer cells resistant to doxorubicin through the miR-221-3p/HIPK2 axis. And miR-221-3p inhibition was confirmed to negate the METTL3-induced breast cancer cell resistance to doxorubicin [46]. The latest study showed that METTL3 promoted HR by regulating the EGF/RAD51 axis, leading to increased doxorubicin resistance in breast cancer cells. Moreover, knockdown of the reader protein YTHDC1 reversed the METTL3-mediated upregulation of epidermal growth factors (EGF) and DNA repair proteins (RAD51). YTHDC1 binds to m6A-modified EGF mRNA and promotes EGF synthesis, suggesting that METTL3 and YTHDC1 together enhance HR and cell survival during doxorubicin treatment, leading to the development of drug resistance in breast cancer [47]. Li et al. found another pathway by which METTL3 contributes to drug resistance in breast cancer. METTL3 increases MALAT1 protein levels in an m6A modification-dependent manner, recruits E2F1 and activates downstream AGR2 transcription to enhance the resistance of breast cancer cells to doxorubicin [48]. In addition, Wu et al. found that ALKBH5 removed the m6A modification of BRCA1 (DNA repair protein), stabilized BRCA1 mRNA and further enhanced DNA repair capacity, which results in reduced efficacy of doxorubicin in breast cancer. Additionally, protein arginine methyltransferase 5 (PRMT5) could enhance the nuclear translocation and translation of ALKBH5. Tadalafil was identified as a novel PRMT5 inhibitor that could increase doxorubicin sensitivity in breast cancer [49]. Wang et al. indicated that FTO activated signal transducer and activator of transcription 3 (STAT3) signaling in breast cancer cells, and both factors worked together to mediate breast cancer resistance to doxorubicin. The knockdown of FTO or STAT3 decreased doxorubicin resistance, which could be a potential strategy in the treatment of breast cancer [50].

### 5-fluorouracil (5-FU) and gemcitabine

5-Fluorouracil (5-FU) and the pyrimidine analog gemcitabine are anti-nucleotide metabolism drugs that are widely used in the treatment of various tumors, especially gastrointestinal tract cancers. Resistance to these drugs is common. There are few studies about the association between drug resistance and m6A methylation, and those that exist mainly focused on pancreatic cancer and colorectal cancer (CRC). The Wnt/β-catenin signaling pathway is genetically conserved and widely involved in embryonic development and tumor development. Tang et al. found that the eraser ALKBH5 removed the m6A modification in the 3’UTR of WIF-1 mRNA to promote its transcription and inhibited Wnt signaling by upregulating Wnt inhibitory factor 1 (WIF-1) instead of regulating β-catenin expression, which makes pancreatic ductal adenocarcinoma (PDAC) cells less sensitive to gemcitabine. ALKBH5 overexpression sensitized PDAC cells to chemotherapy [51]. In addition, Zhang et al. found that the writer METTL14 was associated with gemcitabine resistance in pancreatic cancer cells [52]. They reported that METTL14 was overexpressed in gemcitabine-resistant pancreatic cancer cells and p65 (a transcription factor) promoted the expression of METTL14 and subsequently upregulated cytidine deaminase (CDA), a gemcitabine inhibitor. Silencing METTL14 increased the sensitivity of pancreatic cancer cells to gemcitabine. Another study indicated that METTL3-mediated m6A methylation decreased the expression of lncRNA DBH-AS1 in pancreatic cancer, while DBH-AS1 increased the sensitivity of pancreatic cancer cells to gemcitabine through the miR-3163/USP44 axis. This suggested that METTL3 and DBH-AS1 may be involved in the development of gemcitabine resistance in pancreatic cancer. Targeted regulation of the DBH-AS1 could enhance the efficacy of gemcitabine [53]. ZC3H13 knockdown reduced the translation of PHF10 in a YTHDF1-mediated manner. Dysregulation of PHF10 increased the number of DSBs (DNA double-strand breaks) and inhibited HR. Targeted regulation of ZC3H13 and PHF10 by fisetin inhibited DNA damage repair and enhanced the sensitivity of pancreatic cancer cells to gemcitabine [54]. Liu et al. found that the expression of the oncogene Sect. 62 was regulated by METTL3. Section 62 can maintain the stemness of CRC cells and lead to 5-FU resistance by activating the Wnt/β-catenin pathway. Thus, m6A modification-Sect. 62-β-catenin molecular axis could act as therapeutic targets in improving treatment of CRC [55]. In addition, c-Myc promotes the expression of YTHDF1 in CRC, and knockdown of YTHDF1 enhances the sensitivity of CRC cells to 5-FU [56]. Another study indicated that METTL3-mediated m6A modification increased the expression of miR-181d-5p. This miRNA is contained in exosomes produced by CAFs and can decrease the sensitivity of CRC cells to 5-FU [57].

### Kinase inhibitor (KI)

Targeted therapy, represented by kinase inhibitors, is an indispensable part of current cancer treatment. For example, tyrosine kinase inhibitors (TKIs), such as erlotinib and imatinib, have shown effects in the treatment of lung cancer and leukemia, but the problems of drug resistance and disease recurrence are inevitable. Understanding the mechanism of drug resistance and improving sensitivity to kinase inhibitors are currently a focus. Li et at. found that Notch signaling activation and TUSC7 inhibition occur in erlotinib-resistant lung adenocarcinoma cells [58]. The evolutionarily conserved Notch signaling pathway is related to the survival and proliferation of cancer stem cells [59]. TUSC7 is a long noncoding RNA tumor suppressor whose overexpression can inhibit the proliferation and invasion of tumor cells [60]. They showed that METTL3 persistently activated miR-146a/Notch signaling, while YTHDF2 inhibited TUSC7. Both effects promoted the formation of drug resistance in lung adenocarcinoma cells. The combinational use of TKIs and Notch specific inhibitory lncRNA may pave the way to improve targeted therapy in lung cancer. A previous study found that TKI resistance in leukemia was associated with reduced m6A due to FTO overexpression. In mice, cells with mRNA m6A hypomethylation and FTO upregulation had higher growth rates and were more tolerant to TKIs, while FTO inactivation re-sensitized resistant cells to TKIs [61]. Interestingly, saikosaponin-d (SsD), extracted from Radix Bupleuri, restored the sensitivity of resistant leukemia cells to nilotinib by reversing FTO-mediated m6A hypomethylation, which may provide a new approach for the treatment of acute myeloid leukemia [62]. Another study reported that downregulation of METTL3 and METTL14 decreased imatinib resistance in chronic myelogenous leukemia (CML), thus, inhibitors of METTL3/METTL14 complexes could be a new approach to rescue TKI resistance [63]. In malignant melanoma, METTL3 activates the RAF/MEK/ERK pathway by upregulating EGFR, inducing resistance to PLX4032, a BFAF (V600E) kinase inhibitor. Targeted treatment of METTL3 may improve the efficacy of PLX4032 in patients with advanced melanoma [64]. Chidamide is a histone deacetylase inhibitor (HDACI) that downregulates WTAP and METTL3 to induce c-MET mRNA hypomethylation, reducing the expression of c-MET, which helps to enhance the sensitivity to the ALK/ROS1/c-MET kinase inhibitor crizotinib in the treatment of non-small cell lung cancer (NSCLC) with high expression of c-MET [65]. Significantly, approximately 40% of lung cancer tissues overexpress the MET gene, whereas only 4–6% of lung adenocarcinoma patients have ALK mutations [66, 67]. Sorafenib is a multi-kinase inhibitor that inhibits various kinases, including VEGFR and BRAF. A study has shown that circRNA-SORE induced sorafenib resistance through competitive activation of the Wnt/β-catenin pathway, and the level of circRNA-SORE in sorafenib-resistant hepatocellular carcinoma (HCC) cells was regulated by m6A methylation. Silencing circRNA-SORE could effectively reverse the acquired sorafenib resistance and retard tumor progression [68]. Lin et al. found that METTL3 promoted FOXO3 mRNA stability in a YTHDF1-dependent manner. In sorafenib-resistant HCC cells, the downregulation of METTL3 led to FOXO3 degradation, indicating that sorafenib resistance is mediated by autophagy. Overexpression of FOXO3 rescued the m6A-depedent sorafenib sensitivity in HCC by inhibiting autophagy [69].

### Immunotherapy

Because of the discovery of cancer immune checkpoints and the successful development of immune checkpoint inhibitors, immunotherapy has opened a new era of anticancer treatment that shows promising therapeutic prospects [70]. At present, there are two main types of immune checkpoint inhibitors in clinical application, PD-1/PD-L1 inhibitors and CTLA-4 inhibitors. Despite the unprecedented efficacy of immunotherapy, a large proportion of patients do not benefit from the treatment because of primary and acquired drug resistance. The mechanisms of resistance to immunotherapy are complex and diverse. Recently, the role of m6A modification has attracted attention. Most of the studies on m6A related to immune checkpoint inhibitor resistance have focused on PD-1/PD-L1 inhibitors. METTL3 promotes the back-splicing and circularization of circIGF2BP3 through YTHDC1. This m6A methylation event not only upregulates circIGF2BP3 but also increases the expression of the downstream target gene PKP3. The immunosuppressive effect of PKP3 depends on the deubiquitination of PD-L1 mediated by OTUB1 (a deubiquitinase), which protects PD-L1 from degradation. The accumulated PD-L1 on the surface of NSCLC cells interacts with PD-1 on T cells to decrease T-cell activity. Ultimately, the circIGF2BP3/PKP3/PD-L1 axis allows NSCLC cells to evade the killing effect of CD8^+^ T cells. The inhibition of circIGF2BP3/PKP3 enhanced the treatment efficacy of anti-PD-1 therapy in NSCLC [71]. In addition, inhibition of JNK signal transduction can downregulate METTL3, thereby affecting the stability of PD-L1 mRNA and decreasing its expression. Therefore, the JNK/METTL3/PD-L1 axis is critical for bladder cancer cells to resist death mediated by CD8^+^ T cells. Knockdown of JNK1 or administration of a JNK inhibitor maybe a potential strategy enhancing enhanced immune effect of in bladder cancer [72]. Wang et al. showed that deletion of METTL3 and METTL14 increased the stability of Stat1 mRNA and Irf1 mRNA in a YTHDF2-dependent manner and enhanced IFN-γ-Stat1-Irf1 signaling. It promoted the secretion of IFN-γ, Cxcl9 and Cxcl10 and the recruitment of CD8^+^ T cells in the tumor microenvironment. These cytokines and chemokines enhance the response of pMMR CRC and melanoma to anti-PD-1 therapy, providing a new approach for the combination of immune checkpoint inhibitors and methyltransferase inhibitors in the treatment of CRC and melanoma [73]. In addition to “writers”, “erasers” also affect immunotherapy sensitivity. Yang et al. found that m6A methylation reduced the proliferation and survival rates of melanoma cells, thus exerting a tumor-suppressive effect. FTO-mediated m6A demethylation promoted melanoma growth and reduced the response to anti-PD-1 immunotherapy. Mechanistically, FTO overexpression promoted melanoma development by increasing the key oncogenic genes (PD-1, CXCR4 and SOX10) in mRNA and protein levels and SOX10, and inhibiting IFN-γ-induced melanoma cell death. FTO knockdown increased the sensitivity of melanoma cells to anti-PD-1 therapy [74]. Unlike FTO, which is involved in the regulation of IFN-γ-mediated cytokine and chemokine pathways, ALKBH5 mainly affects the recruitment of immune cells in tumor microenvironment. Li et al. found that ALKBH5 changes the content of lactate in the tumor microenvironment by altering mRNA splicing and the expression of the target gene Mct4/Slc16a3, thereby affecting the recruitment of regulatory T cells (Tregs) and myeloid-derived suppressor cells (MDSCs). Loss of ALKBH5 can enhance the sensitivity of malignant melanoma to anti-PD-1 therapy [75].

### Radiotherapy resistance

As previously mentioned, DSBs repair is one of the causes of radiation resistance. METTL3-mediated m6A modification plays a critical role in the maintenance of glioma stem-like cells (GSCs) and glioma cell dedifferentiation. Silencing METTL3 reduced DSBs repair and increased sensitivity to γ-radiation in GSCs [76]. Another study indicated that the overexpression of ALKBH5 in GSCs enhanced radio-resistance by regulating HR. Knockdown of ALKBH5 significantly reduced the expression of several key genes involved in HR, such as Rad51, XRCC2, BRCA2 and EXO1, while the expression of key genes involved in NHEJ (including Ku70, Ku80 and DNA-PKs) was not affected. This finding demonstrated that ALKBH5 inhibition could be a novel radiosensitizer [77]. In pancreatic cancer, METTL3 modulates the MAPK cascade to increase the resistance of pancreatic cancer cells to chemotherapy and radiotherapy, while knockdown of METTL3 enhances the radiosensitivity of pancreatic cancer cells [39]. In cervical cancer, FTO-mediated demethylation regulates β-catenin expression and promotes ERCC1 activity, which makes cervical squamous cell carcinoma resistant to chemoradiotherapy. ERCC1 (nucleotide excision repair cross-complementation group 1) is a key member of the nucleotide excision repair (NER) system, and its enhanced activity is one of the main reasons for platinum-based drug resistance [78]. In hypopharyngeal squamous cell carcinoma (HPSCC), METTL3 stabilizes the expression of circCUX1 via m6A methylation, while circCUX1 binds to Caspase-1 and inhibits its expression. Caspase-1, also known as interleukin-1β convertase, activates IL-1β and IL-18 and releases them into the extracellular environment to regulate the tumor microenvironment. When tumor cells exist in an inflammatory microenvironment, Caspase-1 actively induces the programmed death of tumor cells. METTL3 reduces tumor cell death through the circCUX1/caspase 1 axis and confers radiation resistance to HPSCC. Knockdown circCUX1 promotes the sensitivity of HPSCC cells to radiotherapy by increasing the release of inflammatory factors [79]. In nasopharyngeal carcinoma (NPC), YTHDC2 increased the translation efficiency and expression of IGF1R mRNA, thereby activating the downstream PI3K-AKT/S6 pathway, inhibiting tumor cell apoptosis, stimulating protein synthesis, and promoting radiotherapy resistance. Knockdown of YTHDC2 promotes the radiotherapy effect in NPC [80].

### Causes of m6A methylation changes due to therapies

Why do these therapies cause alteration of m6A methylation? There are two possible reasons for this. On the one hand, drugs regulate the expression of some m6A genes, thereby affecting methylation levels (direct effect). Sunitinib (TKI) is widely used in the treatment of different types of solid and blood tumors. Ma et al. found that Sunitinib downregulated the expression of FTO, while upregulated the expressions of MELLT14. These changes increased the m6A methylation in vivo [81]. On the other hand, drugs act on a targeted gene directly, then regulate the downstream m6A gene to alter the m6A methylation levels (indirect effect). For instance, Wu et al. found that doxorubicin increased the expression of H2AX and activated m6A modification in breast cancer cells. Doxorubicin enhanced RNA m6A levels through DNA damage [49]. The reason for the changes of m6A methylation levels caused by various therapies is unclear. Further study is needed to understand the detailed mechanisms.

### Role of m6A in cancer

The role of m6A in cancer is reflected in the change of several tumor-related genes. There are two main aspects—one involves the expression of oncogenes, while the other contains the regulation of tumor suppressor genes. Many studies have demonstrated that m6A is critical to tumor initiation, progression and metastasis. He et al. summarized these mechanisms in their review study [82]. On the one hand, m6A as a tumor promoter plays an important role in the development of cancer by promoting the expression of oncogenes and inhibiting the expression of tumor suppressor genes. On the other hand, the suppressing effect of m6A on tumor is reflected in the inhibition of oncogenes and the promotion of tumor suppressor genes. Here we elaborate on recent advances in research of the role of m6A in cancer. Du et al. showed that m6A modification of circ MDK improved its RNA stability, which resulting in the activation of PI3K/AKT/mTOR signaling pathway to promote HCC cell proliferation, migration and invasion [83]. Cui et al. reported that m6A demethylation of LINC00022 by FTO promoted tumor growth of esophageal squamous cell carcinoma [84]. Wang et al. found that YTHDF1 promoted CRC tumorigenesis and metastasis through upregulation of ARHGEF2 translation and protein expression. ARHGEF2 functions to activate RhoA signaling as an oncogene [85]. METTL14 promotes prostate tumorigenesis by inhibiting THBS1 (tumor suppressor gene) expression via an m6A-YTHDF2-dependent manner [86]. YTHDF2 promotes bladder cancer progression by suppressing RIG-I expression, a tumor suppressor related to immune response [87]. Moreover, METTL14 suppresses proliferation and metastasis of CRC by down-regulating oncogenic long non-coding RNA XIST [88]. Li et al. found that METTL3 increased the ZNF677 mRNA stability and promoted its expression. ZNF677 plays a tumor suppressor role in renal cell carcinoma (RCC) through transcriptionally repressing its downstream target CDKN3. METTL3/ZNF677/CDKN3 axis might provide new insight into the potential mechanism of the pathogenesis and development of RCC [89].

## Methods of determining the m6A methylation

A growing number of studies have shown that m6A methylation serve as a potential biomarker for therapy resistant cancer cells [90–92]. Methods of determining the m6A methylation includes LC-MS/MS, Colorimetric and MeRIP-seq. LC-MS/MS can detect the overall m6A level of mRNA by the quantitative and qualitative analysis of the bases. Firstly, Total RNA is isolated from tissues by TRIzol reagent and mRNA and then purified using rRNA depletion kit. Secondly, mRNA is digested by nuclease P1, followed by the addition of NH4HCO3 and alkaline phosphatase with incubation. Lastly, the total amount of m6A in RNA is measured using LC-MS/MS [93]. Colorimetric is a method of measuring the total amount of m6A in RNA, like LC-MS/MS. Compared with LC-MS/MS, colorimetric is more sensitive and convenient by the use of m6A methylation quantification kit [84]. MeRIP-seq identified m6A methylation levels in human with a wide range and high throughput manner conveniently and economically. The m6A methylated mRNA fragments are enriched by immunomagnetic beads with m6A antibody. Then the methylated RNA is purified for further MeRIP sequencing [94].

### Animal model in the m6A research

There are various animal models for m6A research in cancer drug resistance. CDX (Cell-line-derived xenograft) models transplant tumor cells cultured in vitro into mouse and have the advantages of convenience and cost effective. The CDX model mainly used BALB/C-nude, nu/nu, SCID, and NOD-scid mice. The patient-derived xenografts (PDX) models, using human cell lines injected into immunocompromised hosts such as athymic nude mice, are the most widely used models for evaluating cytotoxic therapies against cancer [95]. The specimens used for transplantation are directly derived from human tumor tissue without cultured in vitro, retaining the heterogeneity and growing environment of tumor. Moreover, the common cell lines for exploring the roles of m6A in cancer resistance includes the RCC cell lines (OS-RC-2 and 786–0) [96], NSCLC cell lines (A549 and H1299) [97], ESCC cell lines (Eca109, EC9706, Kyse150, Kyse410, and TE-1) [98], and melanoma cell lines (WM35 and WM115) [74].

## Conclusion

**Fig. 4:**
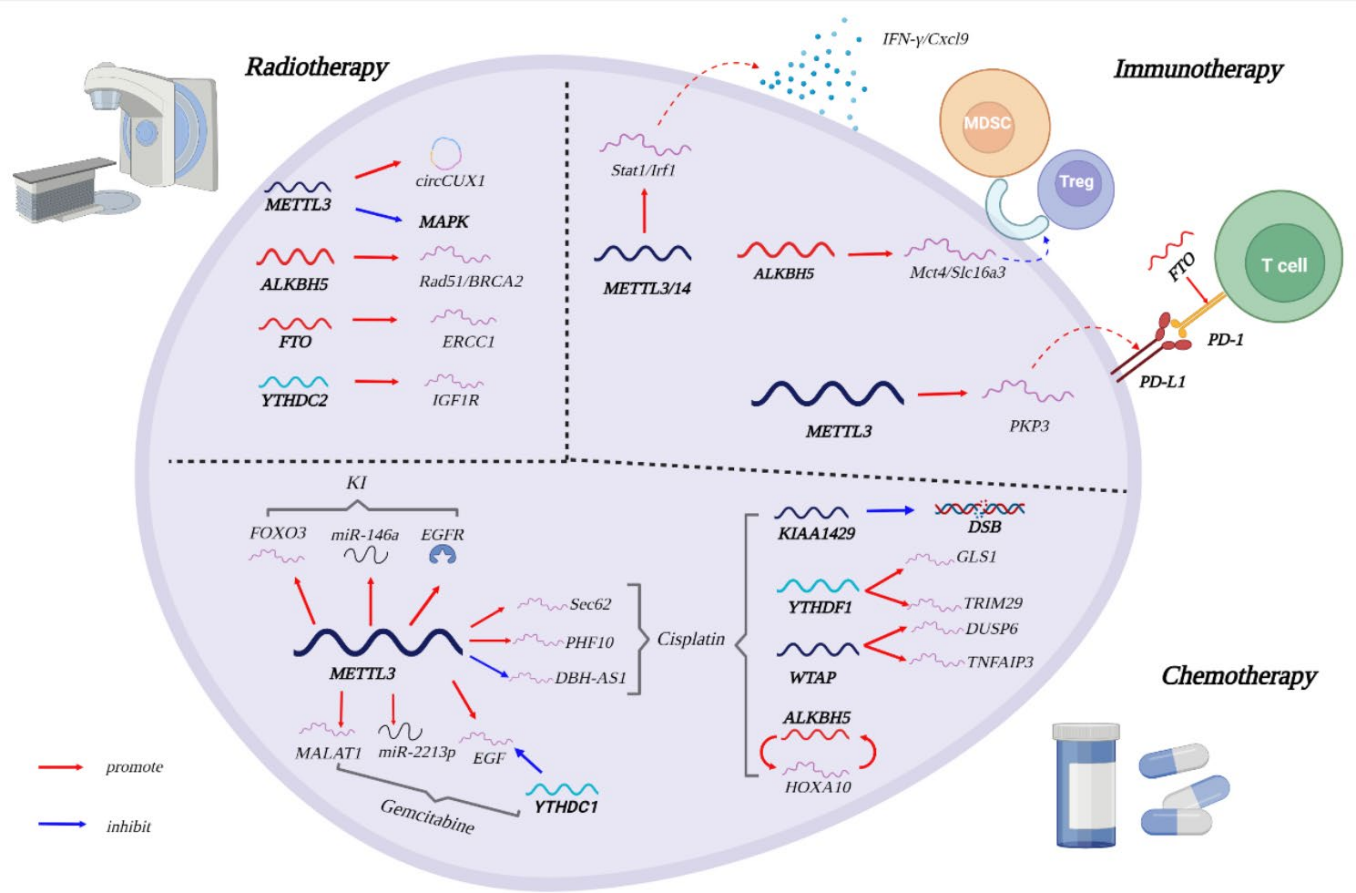
The role of m6A methylation in therapy resistance The m6A regulators promote tumor cell resistance to radiation via stabilizing mRNA, regulating HR and inhibiting signaling pathway. The m6A regulators promote the stability of target gene mRNA and affect the recruitment of immune cell and the secretion of cytokines. The various pathway of m6A regulators in the resistance to chemical drugs (Cisplatin, Gemcitabine, Kinase inhibitor). The red arrows indicate “promote” and the blue arrows indicate “inhibit”

In conclusion, tumor therapeutic resistance is a complex process involving multiple genes, factors and mechanisms that are related to oncogene activation, DNA damage repair, cancer stem cells, hypoxia, tumor microenvironment changes, autophagy, and metabolism. The m6A methylation affects the development of cancer therapy resistance in the above aspects by altering the stability of transcription products of certain key genes and activating or inhibiting certain signaling pathways (Fig. 4; Table 1). Therefore, upregulation or downregulation of certain m6A-related genes and activation or inhibition of certain m6A regulators can enhance the sensitivity of tumors to treatment. Understanding the key role of m6A modification in cancer therapy resistance provides new ideas for the development of drugs or combination therapies.

**Table 1:** The functions of m6A regulators in therapy resistance

Therapy type	Drug	Cancer	Regulator	Regulation	Target/Approach	References
Chemo- therapy	Cisplatin	Ovarian Cancer	ALKBH5	PR	JAK2/STAT3	29
		Bladder Cancer	ALKBH5	PR	CK2	30
		OSCC	ALKBH5	PR	FOXM1/NANOG	32
		Bladder Cancer	WTAP	PR	Circ0008399/TNFAIP3	33
		NKTCL	WTAP	PR	DUSP6	34
		Seminoma	METTL3	PR	ATG5/Autophagy	36
		Seminoma	METTL3	PR	TFAP2C	37
		Seminoma	KIAA1429	PR	DNA damage response	38
		Pancreatic Cancer	METTL3	PR	MAPK	39
		CRC	YTHDF1	PR	GLS1	40
		Ovarian Cancer	YTHDF1	PR	TRIM29	41
		Cholangio-carcinoma	YTHDF2	PR	CDKN1B	43
		Cervical Cancer	YTHDF2	PR	AXIN1	44
	Adriamycin	Breast Cancer	METTL3	PR	miR-221-3p/HIPK2	46
		Breast Cancer	METTL3	PR	EGF/RAD51	47
		Breast Cancer	METTL3	PR	MALAT1/E2F1/AGR2	48
		Breast Cancer	ALKBH5	PR	BRCA1	49
		Breast Cancer	FTO	PR	STAT3	50
	Gemcitabine	Pancreatic Cancer	ALKBH5	PR	WIF-1	51
		Pancreatic Cancer	METTL14	PR	CDA	52
		Pancreatic Cancer	METTL3	PR	DBH-AS1/miR-3163/USP44	53
		Pancreatic Cancer	ZC3H13	PR	PHF10	54
		CRC	METTL3	PR	Wnt/β-catenin	55
		CRC	YTHDF1	PR	c-Myc	56
		CRC	METTL3	PR	miR-181d-5p	57
Targeted-therapy	Erlotinib	Lung- adenocarcinoma	METTL3	PR	miR-146a/Notch	58
		Lung- adenocarcinoma	YTHDF2	PR	TUSC7	58
	TKIs		FTO	PR	-	61
	Imatinib	CML	METTL3/METTL14	PR	-	63
	PLX4032	Melanoma	METTL3	PR	RAF/MEK/ERK	64
	Sorafenib	HCC	METTL3	PR	FOXO3	69
Immuno-therapy	PD-L1	NSCLC	METTL3	PR	circIGF2BP3/ PKP3/OTUB1	71
		Bladder Cancer	METTL3	PR	JNK pathway	72
	PD-1	CRC	METTL3/METTL14	PR	IFN-γ-Stat1-Irf1	73
	PD-1	Melanoma	FTO	PR	PD-1/CXCR4/SOX10	74
	PD-1	Melanoma	ALKBH5	PR	Mct4/Slc16a3	75
Radio-therapy		GBM	METTL3	PR	GSC/DNA repair	76
		GBM	ALKBH5	PR	GSC/HR	77
		Cervical Cancer	FTO	PR	β-catenin/ ERCC1	78
		HSCC	METTL3	PR	circCUX1/Caspase 1	79
		NPC	YTHDC2	PR	IGF1R/ PI3K-AKT/S6	80

OSCC: oral squamous cell carcinoma

NKTCL: nasal-type natural killer/T-cell lymphoma

PR: promote resistance

CRC: colorectal cancer

TKIs: tyrosine kinase inhibitors

CML: chronic myeloid leukemia

HCC: hepatocellular carcinoma

NSCLC: non-small cell lung cancer

GBM: glioblastoma

HR: homologous recombination

HPSCC: hypopharyngeal squamous cell carcinoma

NPC: nasopharyngeal carcinoma

## Data Availability

Not applicable.

## Abbreviations

5-FU: 5-Fluorouracil
ALKBH5: AlkB homologue 5
ATG5: Autophagy related 5
BRCA1: Breast Cancer 1
CAFs: Cancer-associated fibroblasts
CDA: Cytidine deaminase
CDKN1B: Cyclin-dependent kinase N1B
CK 2: Casein kinase 2
CML: Chronic myelogenous leukemia
CSCs: Cancer stem cells
DSBs: DNA double-strand breaks
DUSP6: Dual-specificity phosphatase 6
ECM: Extracellular matrix
EGF: Epidermal growth factor
ERCC1: Excision repair cross-complementing group 1
FOXM1: Forkhead box protein M1
FTO: Fat mass and obesity-related protein
GBM: Glioblastoma
GLS1: Glutaminase 1
GSCs: Glioma stem-like cells
HCC: Hepatocellular carcinoma
HDACI: Histone deacetylase inhibitor
HER2: Human epidermal growth factor receptor 2
HIF-1: Hypoxia-inducible factor-1
HIPK2: Homeodomain-Interacting Protein Kinase 2
HNRNPC: Heterogeneous nuclear ribonucleoprotein C
HOXA10: Homeobox A10
HPSCC: Hypopharyngeal squamous cell carcinoma
HR: Homologous recombination
KIAA1429(VIRMA): Vir-like m6A methyltransferase-associated
KI: Kinase inhibitors
m6A: N6-methyladenosine
MALAT1: Metastasis Associated Lung Adenocarcinoma Transcript 1
MDR: Multidrug resistance
MDSCs: Myeloid-derived suppressor cells
METTL14: Methyltransferase-like 14
METTL3: Methyltransferase-like 3
NER: Nucleotide excision repair
NHEJ: Nonhomologous end joining
NKTCL: Natural killer/T-cell lymphoma
NPC: Nasopharyngeal carcinoma
NSCLC: Non-small cell lung cancer
OTUB1: OTU domain, ubiquitin aldehyde binding 1
PD-1: Programmed cell death 1
PD-L1: Programmed cell death 1 ligand 1
PI3K: Phosphoinositide 3-kinase
RBM15: RNA binding motif protein 15
SsD: Saikosaponin-d
STAT3: Signal transducer and activator of transcription 3
TFAP2C: Transcription factor AP-2 γ
TGF-β: Transforming growth factor-β
TKIs: Tyrosine kinase inhibitors
TME: Tumor microenvironment
TNFAIP3: TNFα-induced protein 3
Tregs: Regulatory T cells
TRIM29: Tripartite motif 29
TUSC7: Tumor suppressor candidate 7
Tβ4: Thymosin β4
VEGFR: Vascular endothelial growth factor receptor
WIF-1: Wnt inhibitory factor 1
WTAP: Wilms tumor 1 associated protein
YTHDC1: YTH domain containing 1
YTHDC2: YTH domain-containing 2
YTHDF1: YTH domain-containing family 1
YTHDF2: YTH domain-containing family 2
ZC3H13: Zinc finger CCCH-type containing 13
